# Is miRNA Regulation the Key to Controlling Non-Melanoma Skin Cancer Evolution?

**DOI:** 10.3390/genes12121929

**Published:** 2021-11-29

**Authors:** Tiberiu Tamas, Mihaela Baciut, Andreea Nutu, Simion Bran, Gabriel Armencea, Sebastian Stoia, Avram Manea, Liana Crisan, Horia Opris, Florin Onisor, Grigore Baciut, Bogdan Crisan, Daiana Opris, Bogdan Bumbu, Adela Tamas, Cristian Dinu

**Affiliations:** 1 Department of Maxillofacial Surgery and Implantology, Faculty of Dentistry, “Iuliu Hațieganu” University of Medicine and Pharmacy, 400012 Cluj-Napoca, Romania; tibi.tamas@yahoo.com (T.T.); mbaciut@yahoo.com (M.B.); dr_brans@yahoo.com (S.B.); garmencea@yahoo.com (G.A.); stoia_sebi@yahoo.com (S.S.); maneaavram@yahoo.com (A.M.); lcrisan@umfcluj.ro (L.C.); horia.opris@gmail.com (H.O.); gbaciut@umfcluj.ro (G.B.); bbcrisan@yahoo.com (B.C.); daiana.a.opris@gmail.com (D.O.); dinu_christian@yahoo.com (C.D.); 2 Research Center for Functional Genomics, Biomedicine and Translational Medicine, “Iuliu Hațieganu” University of Medicine and Pharmacy, 400012 Cluj-Napoca, Romania; andreeanutu.an@gmail.com; 3 Department of Oral Surgery, Dental Medicine, Faculty of Medicine and Pharmacy, University of Oradea, 410087 Oradea, Romania; bogdanbumbu@yahoo.com; 4 Department of Medical Sciences, Pneumology, Faculty of Medicine, “Iuliu Hatieganu” University of Medicine and Pharmacy, 400012 Cluj Napoca, Romania; patcas.adela@yahoo.com

**Keywords:** NMSC, miRNA, skin, cancer, basal, squamous, Merkel, cell, carcinoma, oncogenesis

## Abstract

Non melanoma skin cancer (NMSC) is one of the most common types of skin cancer. It has a number of subtypes, which include basal cell carcinoma, cutaneous squamous cell carcinoma and Merkel cell carcinoma. MicroRNAs are short, non-coding RNA (ribonucleic acid) molecules, capable of regulating gene expression at a post transcriptional level. They play a pivotal role in a variety of physiologic cellular functions and pathologies, including malignant diseases. The development of miRNAs represents an important study field, which has been extensively exploited in melanoma for almost a decade with promising results, therefore we consider it a stepstone for further research projects also in non-melanoma skin cancers. The aim of our study was to explore the current literature in order to present the role of the different miRNAs in some of the most frequent types of NMSC pertaining to oncogenesis, evolution and therapy. The most relevant and accurate available data from the literature were evaluated. Our study concluded that there are almost 100 miRNAs which can be upregulated or downregulated and can play a role in oncogenesis. They can be easily identified in circulation, are stable and they can be important diagnosis/prognosis and therapy monitoring markers.

## 1. Introduction

The skin is the body’s largest organ and one of the first defense lines against various external factors. The maintenance of cellular integrity and underlying complex physiology of the skin are crucial for the overall health of organisms. Exposure to environmental agents including UV(ultraviolet)-radiation or carcinogens could lead to multiple pathological manifestations and compromised cellular integrity. The worldwide incidence of skin cancer is increasing rapidly due to the damage of the ozonosphere [1,2,3,4,5,6,7].

Malignant skin tumors are largely divided into melanoma and non-melanoma cancers. Non melanoma skin cancer (NMSC) is one of the most common types of skin cancer. According to the Globocan statistical data, there were over 1 million new cases worldwide in 2020, placing NMSC on the 4th place among all malignancies. Although the mortality is lower than other types of cancer, there were more than 60,000 cases reported in 2020 [8]. It has a number of subtypes, which include basal cell carcinoma (BCC), cutaneous squamous cell carcinoma (SCC) and Merkel cell carcinoma (MCC). BCC is the most frequent cutaneous carcinoma, followed by SCC and accounts for more than 20% of all skin cancer cases all over the world [9,10,11,12].

MicroRNAs (miRNAs) are short, non-coding RNA molecules formed by 17 to 23 nucleotides (nt). These molecules have the ability of regulating gene expression in post transcriptional phase. Being encoded within exons and introns, they play a major role in various physiological cellular functions and pathologies, including cancer [13,14,15,16,17,18].

Their discovery by two research groups in 1993 was one of the greatest achievements in molecular biology [19,20]. MiRNA biogenesis is a progressive process beginning with the production of primary miRNAs (pri-miRNAs) by specific *DNA* (deoxyribonucleic acid) transcripts which are transformed into precursor miRNAs (pre-miRNAs) followed by mature miRNAs. MiRNAs are able to regulate not only gene expression, but also protein translation. They are actively secreted into the bloodstream by a variety of cancer cells. MiRNAs can be identified in tissue samples and biological fluids such as cerebrospinal fluid, serum, plasma, saliva and urine. Usually, the circulating miRNAs are the first choice in the clinical setting due to the simplicity of technique but the result can be less concludent than the ones from tissue samples [21,22,23,24,25,26,27].

**Figure 1:**
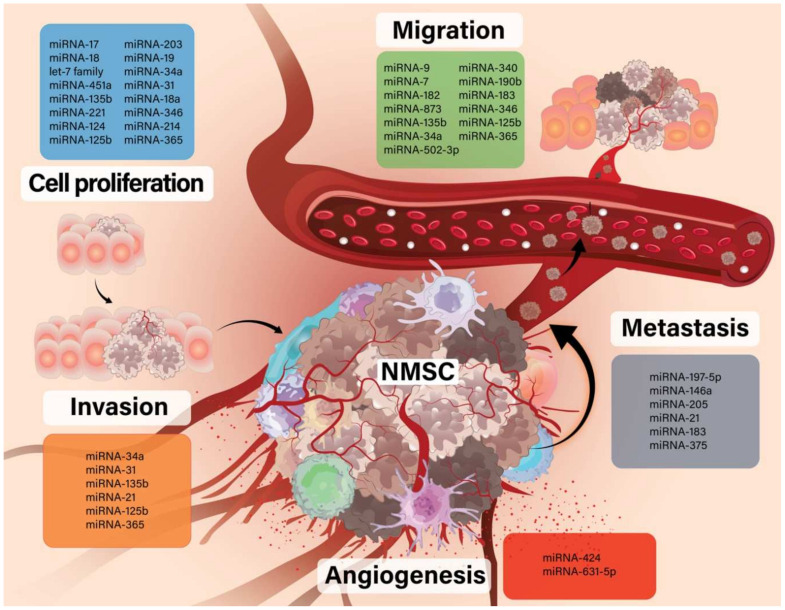
The role of different miRNA in NMSC evolution.

The aim of our study was to explore the current literature in order to present the role of the different miRNAs in some of the most frequent types of NMSC pertaining to oncogenesis, evolution and therapy (Figure 1).

## 2. Materials and Methods

In the present review we encompassed the most relevant and accurate available data from the literature regarding the role of miRNAs mechanisms in oncogenesis, tumor progression, therapy and drug response in NMSC. The following keywords were searched: “miRNA”, “non-melanoma”, “skin”, “cancer”. Our process of selection included two of the most reliable databases in the medical field, EMBASE and PubMed, under specified criteria such as 10-years filter English language. The final article selection remained subjective. After the selection of the articles we presented the miRNAs function and their implications of miRNAs in different types of NMSC.

## 3. miRNA Function in Oncogenesis, Evolution and Therapy 

miRNAs are small noncoding RNAs (17–23 nucleotides) expressed endogenously that occur both intra- and intergenically. The primary miRNAs are transcribed by RNA polymerase II resulting in long transcript forms which are nuclear cleaved by the Drosha RNase III endonuclease in a 60–70 nt precursor miRNA (pre-miRNA). The pre-miRNA is further processed by the RNase III endonucleproliferationase Dicer, resulting in a miRNA: miRNA duplex. One strand of the miRNA: miRNA duplex, the mature miRNA, is loaded into the RNA-induced silencing complex (RISC), while the other strand is degraded. The binding of the miRNA-RISC complex induces a translational repression or destabilization of mRNA. 

**Figure 2:**
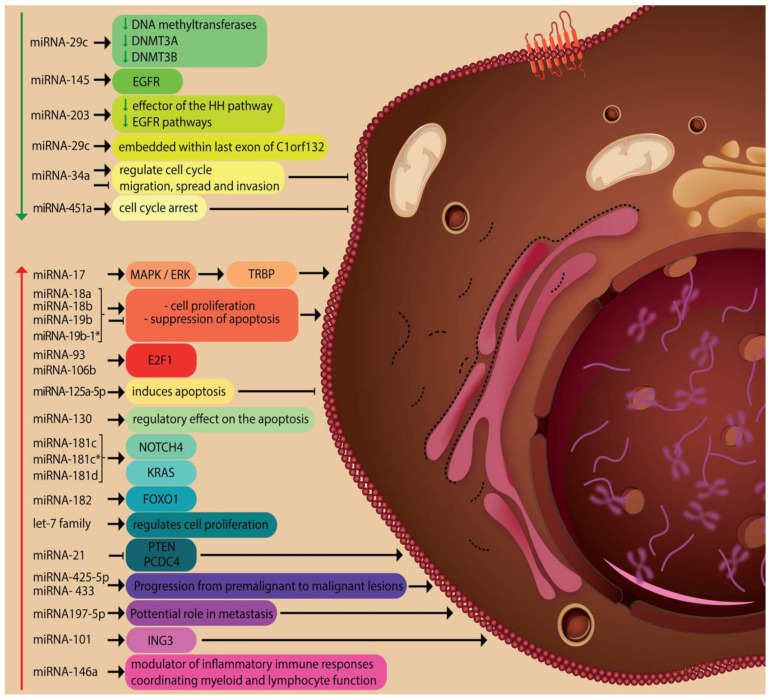
Physiological and pathological processes involving miRNAs.

Through this action, miRNAs are involved in many physiological and pathological processes being recognized as potential therapeutic targets as well as possible biomarkers with diagnostic and/or prognostic potential (Figure 2). There is also a series of unconventional roles attributed to these molecules. Thus, miRNAs can activate Toll-like receptors displaying a pro-inflammatory/pro-metastatic potential, and hence, specific miRNAs can become future therapy targets. Also, miRNAs can act on protein expression in a cell cycle-dependent manner [28,29,30,31,32,33].

### 3.1. The Role of miRNAs in Basal Cell Carcinoma (BCC)

BCC is the most commonly encountered invasive skin cancer worldwide, arising from the basal skin cell layer. Metastasis of BCC is very rare, but the metastasized disease has a poor prognosis [34,35]. 

Regarding the role of miRNA in BCC, relevant contributions were brought by Sand M. et al. [13,14], as well as Heffelfinger et al. [36]. According to these authors, miRNA-21, miRNA-143, miRNA-148a, miRNA-378, miRNA-182, and let-7 family members were the most highly expressed in these tumors. 21 miRNAs have shown particular difference in expression between nodular and infiltrative BCCs. Sixteen significantly up-regulated miRNAs (miRNA-17, miRNA-18a, miRNA-18b, miRNA-19b, miRNA-19b-1*, miRNA-93, miRNA-106b, miRNA-125a-5p, miRNA-130a, miRNA-181c, miRNA-181c*, miRNA-181d, miRNA-182, miRNA-455-3p, miRNA-455-5p and miRNA-542-5p) and ten significantly down-regulated miRNAs (miRNA-29c, miRNA-29c*, miRNA-139-5p, miRNA-140-3p, miRNA-145, miRNA-378, miRNA-572, miRNA-638, miRNA-2861 and miRNA-3196) were detected in BCC compared with normal skin. 

Sonkoly E et al. found in 2012 that miRNA-203 functions as a tumor suppressor miRNA in BCC. Reduced levels of miRNA-203 expression in BCCs are likely to sustain a high proliferative rate. It is known that upregulation of the Hedgehog (HH) pathway is the main molecular anomaly in all BCCs. Evidence suggests that aberrant activation of HH signaling is sufficient to initiate BCC carcinogenesis. MiRNA-203 functions as a downstream effector of the HH pathway and the epidermal growth factor (EGFR) pathways. They also mentioned that miRNA-203 may be a potential therapeutic target for the treatment of BCC [37].

Another study of Sand and colleagues identified 33 upregulated miRNAs in BCC by next-generation sequencing under neoadjuvant vismodegib therapy [38]. 

Al-Eryani L and collaborators noted that miRNA-425-5p and miRNA-433 were elevated in BCCs and other six miRNAs (miRNA-29c, miRNA-381, miRNA-452, miRNA-487b, miRNA-494 and miRNA-590-5p) were specifically inhibited [1,39].

Hu P et al. described the role of miRNA-34a present in serum samples of BCC patients. They observed low levels of miRNA-34a expression in patients having BCC relative to the healthy population and they established a correlation between miRNA expression with tumor cell diameter, lymph node metastasis and histological types of BCC. In the low expression group, median progression-free survival, overall survival time and rate were significantly improved. Also, the prognosis of basal cell carcinoma patients with low expression levels of miRNA-34a was poor [1,40].

Sun and Jiang confirmed miRNA-451a as a BCC tumor suppressor miRNA. In their study, miRNA-451a expression was reduced and the overexpression of miRNA-451a suppressed BCC cell growth through G1 cell cycle arrest, suggesting a therapeutic target of miRNA-451a in BCC [41].

Chang et al. studied the role of miR197-5p and they found that the miRNA was expressed in all patients with metastatic basal cell carcinoma (MBCC) and non-metastatic basal cell carcinoma (non-MBCC). Addition of a synthetic inhibitor of miRNA-197 significantly reduced fibroblast migration but not invasion. This means that miR197-5p is a potential candidate for future research into mechanisms of BCC metastasis [1,42].

Wan and Li found many dysregulated miRNAs, such as miRNA-29b, miRNA-7b, miRNA-141 miRNA-9, miRNA-203, miRNA-200a, miRNA-7c and miRNA-132, and that these miRNAs were significantly associated with BCC. MiRNA-203, miRNA-495, miRNA-385, miRNA-220a and miRNA-30e target *PTCH1*, which is an important component of tumor deriving signaling in BCC pathophysiology. Their network analysis identified some miRNAs such as miRNA-101, miRNA-103, miRNA-130a, and that miRNA-144 directly targeted the *inhibitor of growth protein 3 (ING3)*, a tumor suppressor protein that interacts with tumor suppressor *TP53* [1,43].

MiRNA-146a is of particular interest in the etiology of NMSCs, as it is an important modulator of inflammatory immune responses, coordinating myeloid and lymphocyte function to impact aspects of immunity. MiRNA-146a has 224 potential mRNA binding targets on the cancer susceptibility gene *RNASEL* (Ribonuclease L). In their study, Farzan et al. found that miR146a may play a role in the development of NMSC, both BCC and SCC [44] (Table 1).

### 3.2. The Implication of miRNAs in Squamous Cell Carcinoma (SCC)

The cutaneous SCC is a skin tumor originated from epidermal keratinocytes and the second most common skin cancer with arising incidence [34,45].

Exposure to the ultraviolet radiation is the main risk factor for SCC development. UVR induces a significant accumulation of *DNA* damage in the cutaneous cells, providing a wide mutational landscape which drives to SCC carcinogenesis. Other risk factors are old age, immunosuppression, smoking and genetic factors [46,47].

In the latest years, there have been major improvements in the recognition of target genes and the functions of numerous miRNAs [48].

Sand et al. published a series of works regarding the role of miRNA in SCC. They found in 2010 that the expression levels of the miRNA machinery, namely Drosha, DGCR8, AGO1, AGO2, PACT, and TARBP1, were significantly higher compared to healthy controls and Dicer levels were significantly higher compared to intra-individual controls [49,50]. They also performed a comparative miRNA analysis between SCC biopsies and adjacent healthy skin. Their result identified 13 particularly elevated and 18 downregulated miRNAs in SCC tumors. MiRNA-31 was expressed as higher upregulated miRNA in SCC in comparison with normal skin [34,51].

Dziunycz et al. identified in SCC the elevated expression of miRNA-21 and miRNA-184 and the lower expression of miRNA-203. Also, they described the increased expression of miRNA-21, hsamiR-203, and miRNA-205 by UVA exposure, in comparison with UVB exposure, which increased miRNA-203 and decreased miRNA-205 [14,52].

Yamane et al. identified the downregulation in SCC both in vitro and in vivo of miRNA-124 and miRNA-214. They are regulators of extracellular-signal-regulated kinase 1, 2 and 3 [14,53].

MiRNA-205 was associated with particular pathological characteristics of poor prognosis, including desmoplasia, perineural invasion and infiltrative patterns, clinically correlated with local recurrence. On the other side, miRNA-203 expression was linked to a favorable prognosis due to its identification mainly in well-differentiated areas and rarely in the invasion site [27,54,55].

Zhang et al. correlated low levels of miRNA-20a with poorer overall survival, proving the reduced expression of miRNA-20a in association with worse TNM (tumor node metastasis) staging [27,56].

Gong et al. demonstrated that miRNA-221 plays an oncogenic function in SCC. miRNA-221 expression was significantly higher in SCC tissues than in normal tissues. miRNA-221 knockdown inhibited the proliferation and cell cycle, while upregulation of miRNA-221 presented the opposite role. *PTEN* is a direct target gene of miRNA-221 [57]. 

Mutations to the TP53 gene are common in UV-exposed keratinocytes and contribute to apoptotic resistance in skin cancer. MiRNA-34a influences *TP53* action on several cell processes such as growth arrest, senescence and apoptosis, also recognized as a target of P53. Therefore, in non-melanoma skin tumors, the key of overcoming resistance to cell death signaling could be represented by targeting *TP53* gain-of-function mutations. Lefort et al. found that in SCC miRNA-34a expression was downregulated both in vivo and in vitro [58,59,60,61,62,63]. 

Kanitz et al. associated lower levels of miRNA-361-5p in SCC in comparison with normal skin and identified *VEGFA* (Vascular Endothelial Growth Factor A) as a direct target of miRNA-361-5p [64]. 

Chen et al. demonstrated that miRNA-346 is downregulated and can function as an onco- miRNA in SCC [65].

MiRNA-125b has been found by Xu et al. to be downregulated in premalignant lesions (actinic keratosis) and in SCC in comparison with normal tissue. It has a potential role of therapeutic biomarker. *Matrix metalloproteinase (MMP)13* was identified as a direct target of miRNA-125b [66].

Olasz et al. identified in SCC an oncogenic role of miRNA-135b, with *leucine zipper tumor suppressor 1 (LZTS1)* as its direct target. In early stages of SCC progression, MiRNA-135b can influence cell migration and tumor invasiveness, acting as an oncogenic miRNA in human keratinocytes. By in vitro analysis, downregulation of *LZTS1* mRNA was associated with miRNA-135b overexpression and also with increased mobility and aggressivity of malignant cells [67]. 

In the study of Zhou M. et al. the authors demonstrated that miRNA-365 was overexpressed both in fresh SCC samples and paraffin-embedded tissues and that miRNA-365 may act as an onco-miRNA in cutaneous SCC both in vitro and in vivo. These findings demonstrate that miRNA-365 may be a carcinogenic factor in cutaneous SCC and a potential target in cutaneous SCC therapy. The overexpression of miRNA-365 in cutaneous SCC can be used as a potential indicator both in the clinical diagnosis and treatment [68] (Table 2).

### 3.3. Modified miRNAs in Merkel Cell Carcinoma (MCC)

Merkel cell carcinoma (MCC) is a highly malignant non-melanoma skin tumor which usually arises in the sun-exposed skin [69,70]. A very high percent of the MCC is also associated with immune deficiencies and the presence of Merkel cell polyomavirus (MCPyV) [71,72]. 

The miRNAs role in MCC was studied by several authors. As compared to other variants of NMSCs, Ning et al. have found the overexpression of eight miRNAs (miRNA-502-3p, miRNA-9, miRNA-7, miRNA-340, miRNA-182, miRNA-190b, miRNA-873, and miRNA-183) and the suppression of three (miRNA-3170, miRNA-125b, and miRNA-374c), with the abundance of miRNA-182 in the malignant cells. By concomitent evaluating the expression of four miRNAs (miRNA-182, miRNA-183, miRNA 190b, and miRNA-340) in the MCPyV-negative cell line noted their downregulation. The authors concluded that these miRNA have a possible diagnostic role in cases of MCPyV-positive MCC [73]. 

Renwick et al. have correctly discerned BCC from MCC, due to overexpression of miRNA-205 and miRNA-375 [74]. In 2019 it was found that *Atonal homolog 1 (ATOH1)* expression, a tumor suppressor gene, binds and further activates miRNA-375, the highest abundant miRNA in MCC. In experimental models, it was shown that ATOH1 knockdown in cell lines reduced miRNA-375 expression. ATOH1 overexpression increases the risk of metastasis. The infection with MCPyV promotes carcinogenesis by ATOH1 stimulation [75].

Vieja et al. described the miRs related to positivity and negativity of MCPyV in MCCs. Therefore, miRNA-30a, miRNA-34a, miRNA-142-3p, and miRNA-1539 were overexpressed in MCPyV-positive MCCs, while miRNA-181d was overexpressed in MCPyV-negative MCCs [76].

Another important finding was the clinical possibility of associating circulating miRNAs as biomarkers for MCC. Using RT-PCR method, Moens et al. evaluated the expression of several miRNAs in exosomes and identified the presence of miRNA-30a, miRNA-125b, miRNA-183, miRNA-190b, and miRNA-375 [77]. Tuaeva et al. translated 11 of 22 circulating tumor DNA and circulating miRNAs into clinical practice to predict the clinical-pathological features of tumors thus ameliorating the diagnostic and therapeutic strategies available for cancer patients [78] (Table 3).

## 4. Discussion

Despite the advances in the prevention, diagnosis and treatment of NMSC worldwide, the exact molecular basis of NMSC remains unclear. miRNAs are considered to be important genetic regulators of various biological processes, including cell proliferation, development, invasion and apoptosis and can exhibit a dual function of either pro- or antitumoral action. 

A number of previous studies have demonstrated that miRNAs are involved in several pathologies, especially in different types of cancer. MiRNAs were brought in the spotlight by the intense research made in this domain due to their implication in the tumorigenesis mechanism. For example, miRNA-17-92 with its subtype miRNA-17-15p expresses an oncogene function involved in tumor aggressiveness in several malignancies such as colorectal, liver and gastric cancer. On the other hand, the same miRNA can exert a tumor-suppressive effect in breast, prostate and lung cancer [79,80].

Lately, the Next-Generation Sequencing technologies (NGS) have offered a new opportunity to identify different miRNAs sequences that play a crucial role in the regulation of several cellular processes, such as transcription, post-transcriptional modifications, and signal transduction. Several published reviews have shown a wide range of miRNAs that play an important role in tumorigenesis, tumor progression and in drug response [81,82,83,84].

In the latest years, miRNAs have been under the spotlight due to their potential role of therapeutic targets. In vivo studies on different mice cell disease lines have proved the efficacy of miRNA targeting therapeutic strategies. Nowadays, RNA molecules are being clinically studied as therapeutic molecules, mostly for systemic application. For in vivo applications, multiple modifications are used in combination to achieve the best functional efficiency and stability [34,85,86,87].

In the field of skin cancers, miRNA have been broadly studied mostly in melanoma but also in other tumor types [88]. Due to the tumoral heterogeneity which determines highly aggressive characteristics and negative prognostic factors, melanoma has represented an intense study field for miRNAs. According to the miRNA expression in tumor tissue or in body fluids, a few years ago the role of miRNA-221 as prognostic factor was one of the first identified [89]. Furthermore, the expression of other miRNAs such as miRNA-203, miRNA-10b, miRNA-200b, miRNA-155 have been associated with metastasis and negative outcome. Potential therapeutic targets such as miRNA-675, miRNA-204, the combination between miRNA-135 and large tumor suppressor kinase 2 (LATS2) have also been suggested [90,91,92]. As for prognostic biomarker panel of shorter survival, miRNA-125b, miRNA-200c and miRNA-205 have been proposed [93]. Larger studies involving the analysis of over 25 miRNAs have correlated the presence of miRNA-10b, miRNA-16 and miRNA-21 with poor prognosis [94]. Another role for miRNAs in melanoma patients is the involvement in the therapy resistance process and particular miRNAs such as miRNA-92a-1-5p, miRNA-708-5p in association with specific genes have been identified [95]. 

In patients who undergo targeted therapy with *BRAF* (v-raf murine sarcoma viral oncogene homolog B1) and *MEK* (mitogen-activated protein kinase) inhibitors the expression of downregulated miRNA-579-3p is associated with treatment resistance, but miRNA-126-3p is responsible for increasing the sensitivity in dabrafenib-resistant patients [96,97]. Chemosensitivity of the malignant cells was correlated with the expression of miRNA-211 but the sensitivity to MAPK inhibitors is decreased when miRNA-214 is overexpressed [98,99]. Referring to immunotherapy in melanoma, the most innovative treatment option implemented so far, miRNAs can be further used as blood markers for efficacy by measuring their plasmatic levels before starting the treatment [100]. 

Increasing numbers of miRNAs have been identified as critical regulators in the initiation and progression of NMSC. In this study we have selected three of the most frequent types of NMSC and the role of miRNA was presented individually. Regarding BCC, studies have shown that more than 60 miRNAs were identified and can play a role in diagnosis or treatment. 

The BCC remains the most frequent type of NMSC. The literature data identified numerous types of miRNAs associated with the malignancy and more particularly, with the variation between the nodular and infiltrative BCCs. A particular type of miRNA, miRNA-203 is involved in the carcinogenesis process by exerting a downstream effectory function upon the Hedgehog pathway and *EGFR* (epidermal growth factor receptor) and could represent a possible therapeutic target in this setting. Also, numerous types of miRNAs were associated with other mechanisms of the malignant cycle such as *PTCH1* (patched 1) and *ING3* (inhibitor of growth factor family 3). Studies have evidenced a possible role upon tumor progression and metastasis and miR197-5p was the main type identified. Another possible biomarker was miRNA-34a, associated with poor prognosis characteristics in BCC patients. MiRNA-146a has gained particular attention due to its effect on the inflammatory immune response and its common role in both BCC and SCC tumor development. Even if the prognosis is not severe and metastases are rare, some cases of advanced BCC or subtypes of BCC like the morpheus type are associated with a very poor prognosis. In our opinion, a further direction of research could be represented by the differentiation of BCC subtypes according to the miRNAs expression. The results could provide a better treatment selection and improve patients care delivery.

Many miRNAs are upregulated or downregulated in SCC and MCC and they play a role in the evolution of these pathologies. As we already know, the presence of regional metastasis in SCC and MCC is more frequent than in BCC and can have a poor prognosis. In SCC, distinct types of miRNAs were influenced and associated with either UVA or UVB exposure and several specific types were correlated to the malignant process in comparison with the expression in the normal skin, out of which miRNA-31 was the most common. Apart from their negative predictive role due to carcinogenesis and tumor progression, particular types were linked to a favorable prognosis such as miRNA-203. MiRNAs can also express their inhibiting or stimulating role in the malignant process by targeting specific genes such as *PTEN*, *TP53*, *VEGFA*, *MMP13* and *LZTS1* suggesting a synergic mechanism of action. Another miRNA with oncogene role intense studied in vivo and in vitro was miRNA-365 with a potential role in the clinical setting. 

In MCC, the study of miRNA has shown the association between particular types of miRNA and their correlation with the MCPyV-positive subtype of MCC. Also, previous studies highlighted distinct types of miRNAs linked to MCPyV-positive and MCPyV-negative MCC, suggesting their different prognostic role in this particular setting. The influence of miRNA in MCC carcinogenesis was integrated either by downregulation, overexpression or correlation to *ATOH1* tumor suppression gene. The potential role of miRNA as a biomarker in MCC was evidenced by RT-PCR (reverse transcription polymerase chain reaction) of exosomes and by circulating DNA and miRNA and could provide a key to personalized therapy in this category of patients.

Understanding the role of miRNAs in the diagnosis and metastasis process could increase the survival rate in this category of patients. The method of identification can vary from tissue sample, which can present disadvantages due to the biopsy failure, to circulating miRNAs from body fluids. The latter mechanism involves a non-invasive technique, which can be easily repeated and provides real-time monitoring, but its sensitivity can vary. Still, this method can be used for early detection as in comparison with the tissue sample, which is characterized by a higher sensitivity because the miRNAs are originated only from the tumor cells [101,102]. Introducing the liquid biopsy in the clinical daily practice can become a preferred diagnosis tool due to the less invasive characteristic and in combination with dermoscopy could differentiate the aggressivity profile types of NMSC. 

Regardless the type of sample, miRNAs can be identified by the following technologies: microarray platforms which provide the characteristics of the detected miRNAs, quantitative real-time polymerase chain reaction (qPCR), quantitative reverse transcription real-time PCR (RT-qPCR) with different versions, used mainly for malignant biomarker identification. Nevertheless, RT-PCR is the gold standard tool for diagnosis and research in this field [103,104,105]. Furthermore, apart from the traditional techniques mentioned above, recent studies suggest the use of next-generation sequencing (NGS), miRNA enzyme immunoassay (miREIA) and multiplexed miRNA analysis which need further investigation in skin cancer patients in order to be broadly validated for clinical use and replace the actual gold standard method of detection [106,107,108]. 

The development of miRNAs represents an important study field, which has been extensively exploited in melanoma for almost a decade with promising results, therefore we consider it a stepstone for further research projects also in non-melanoma skin cancers which provide so far limited results. 

Probably the highest impact upon treatment and prognosis could be represented by the early identification of the patients with high risk of developing metastasis. This work-up could offer a personalized treatment approach with a better prognosis and improved overall survival.

## 5. Conclusions

Non melanoma skin cancers need constant attention due to their increasing incidence and rapid evolution. Discovering molecular mechanisms helps us understand tumorigenesis, the process of metastasis and evolution while also leading to development of new therapeutic strategies. The miRNAs, these small non-coding RNAs that control gene expression at the post transcriptional level, are gaining increasing attention. There are almost 100 miRNAs which can be upregulated or downregulated and can play a role in oncogenesis. They can be easily identified in circulation, are stable and they can be important indicators of therapeutic evolution, diagnosis and prognosis. A reliable technology for assessing miRNAs, especially in the clinical workflow, is also mandatory. The miRNAs impact on the chemosensitivity and chemoresistance in tumors can be a pathway for new researches and could provide new strategies to optimize therapeutic approaches. 

Even if immunotherapy has revolutionized the therapeutic approach of advanced NMSC, a high percent of the patients do not respond to this therapy, and their prognosis is severe with a very low survival rate. 

Unfortunately, selecting patients who have a real clinical benefit from this therapy from those who do not improve is not yet currently available. Using miRNAs could allow for individualized treatments and better responses to chemotherapy treatments and immunotherapy.

## Figures and Tables

**Table 1:** Modified miRNAs in BCC.

Nr	Ref	Expression	miRNA	Probe	Species	Role
1	Sand M. et al. [13,14]	upregulated	miRNA-17	tissue	human	pro-growth miRNA regulated in vitro by MAPK (mitogen-activated protein kinase)/ ERK-induced phosphorylation of TRBP (TAR-RNA binding protein)
			miRNA-18a miRNA-18b			cell proliferation and the suppression of apoptosis
			miRNA-19b miRNA-19b-1*			responsible for enhanced cell pro- liferation and the suppression of apoptosis
			miRNA-93			transcription factor E2F1 (E2 promoter binding factor 1) is a target gene of miRNA-93
			miRNA-106b			transcription factor E2F1 is a target gene of miRNA-106b
			miRNA-125a-5p			induces apoptosis
			miRNA-130a			regulatory effect on the apoptosis
			miRNA-181c miRNA-181c* miRNA-181d			targets *NOTCH4*( neurogenic locus notch homolog-4) and *KRAS* (Kirsten rat sarcoma virus)
			miRNA-182			negatively regulate human *Forkheadbox* *O1* (FOXO1)
			miRNA-455-3p miRNA-455-5p miRNA-542-5p			not mentioned
		downregulated	miRNA-29c			downregulates *DNA* methyltransferases DNMT3A and DNMT3B
			miRNA-29c* miRNA-139-5p miRNA-140-3p			not mentioned
			miRNA-145			targets *EGFR*
			miRNA-572 miRNA-638 miRNA-2861 miRNA-3196			not mentioned
2	Heffelfinger et al. [36]	upregulated	let-7 family	tissue	human	involved in regulating cell proliferation
			miRNA-21			represses a variety of tumor suppressors such as *PTEN* (Phosphatase And Tensin Homolog) and PCDC4 (Programmed cell death protein 4)
			miRNA-148a miRNA-143 miRNA-378			not mentioned
3	Sonkoly E et al. [37]	downregulated	miRNA-203	tissue	human	downstream effector of the HH pathway and EGFR pathways. Potential therapeutic target for the treatment of BCC
4	Al-Eryani L et al. [39]	upregulated	miRNA-425-5p	tissue (arsenic induced lession)	human	Premalignant lesions progression to malignancy
			miRNA- 433			
		downregulated	miRNA-29c			encoded in the last exon of C1orf132 (chromosome 1 open reading frame 132), and the transcript converts an unknown open reading frame
			miRNA-381 miRNA-452 miRNA487b miRNA-494 miRNA-590-5p			not mentioned
5	Hu P et al. [40]	downregulated	miRNA-34a	blood	human	can regulate cell cycle and inhibit the migration, spread and invasion of tumor cells
6	Sun H, Jiang P [41]	downregulated	miRNA-451a	tissue	human & mouse	limits cell proliferation by cell cycle arrest induction, suggesting the potential therapeutic target of miRNA-451a in BCC
7	Chang J et al. [42]	upregulated	miRNA197-5p	blood	human	Potential role in metastasis process
8	Wan C, Li Y [43]		miRNA-101	tissue	human	targets *ING3*
			miRNA-7b miRNA-141 miRNA-9 miRNA-200a miRNA-203 miRNA-7c miRNA-132 miRNA-203 miRNA-495 miRNA-385 miRNA-220a miRNA-30e miRNA-29b miRNA-103 miRNA-130a miRNA-144			not mentioned
9	Farzan SF et al. [44]	upregulated	miRNA-146a	blood	human	modulator of inflammatory immune responses, coordinating myeloid and lymphocyte function to impact aspects of both innate and adaptive immunity

**Table 2:** Modified miRNAs in SCC.

Nr	Ref	Expression	miRNA	Probe	Species	Role
1	Sand M. et al. [51]	upregulated	miRNA-31	tissue	human	downregulate the tumor suppressor RhoBTB1 (Rho Related BTB Domain Containing 1) in the cSCC cell line A-431, determing cell proliferation and invasion
			miRNA-135b			miRNA-135b can regulate cell migration and tumor invasiveness in early stages of SCC progression and can act as an oncogenic miRNA in human keratinocytes
			miRNA-424			determines angiogenesis, regulates cell-autonomous angiogenic functions
			miRNA-21* miRNA-374a miRNA-196a			not mentioned
			miRNA-18a			associated with the Sonic Hedgehog pathway, correlated with molecular pathogenesis of cSCC
			miRNA-766 miRNA-128			not mentioned
			miRNA-130b			downregulate the tumor suppressor protein 53-induced nuclear protein 1 (TP53INP1)
			miRNA-455-5p			not mentioned
			miRNA-21			targets phosphatase and *tensin homolog (PTEN)*, *PDC4 (Programed Cell Death 4)* and *BTG2* (B-cell translocation gene 2)
		downregulated	miRNA-30a* miRNA-133b miRNA-101 miRNA-4324 miRNA-136			not mentioned
			miRNA-378			targets insulin-like growth factor 1 receptor (IGF1R) and caspase 3; reduced expression in basal cell carcinoma
			miRNA-204 miRNA-497 miRNA-29c miRNA-214			not mentioned
			miRNA-145			inhibits actin-binding protein Fascin homolog 1 (FSCN1) in esophageal squamous cell carcinoma; down-regulated in basal cell carcinoma
			miRNA-199a-5p miRNA-125b			not mentioned
			miRNA-140-3p			targets CD38; down-regulated in basal cell carcinoma
			miRNA-26a			downregulation of oncogene *Histone-lysine N-methyltransferase (EZH2)*
2	P Dziunycz et al. [52]	upregulated	miRNA-21	tissue	human	essential role in the development or maintenance of SCC of the skin
			miRNA-184 miRNA-205			not mentioned
		downregulated	miRNA-203			unleash p63 expression, leading to decreased cell senescence and supporting SCC formation
			miRNA-378			not mentioned
3	Yamane et al. [53]	downregulated	miRNA-124 miRNA-214	Tissue and serum	human	lead to overexpression of ERK1/2. May lead to the development of useful biomarkers for early detection of this tumor and to new treatments using miRNA
4	Zhang L et al. [56]	downregulated	miRNA-20a	tissue	human	might play important roles in the tumorigenesis and progression of CSCC patients, may serve as a novel molecular marker to predict the tumor progression and inferior prognosis of CSCC patients
5	Gong et al. [57]	upregulated	miRNA-221	blood	human	significantly promotes cell proliferation
6	Kanitz et al. [64]	downregulated	miRNA-361-5p	tissue	human	regulator of *VEGFA* expression
7	Chen et al. [65]	downregulated	miRNA-346	tissue	human	promotes the cSCC cell proliferation and migration through directly targeting *SRCIN1* (SRC Kinase Signaling Inhibitor 1). This study may provide a new therapeutic target for cSCC.
8	Xu et al. [66]	downregulated	miRNA-125b	tissue	human	potential therapeutic biomarker. *Matrix metalloproteinase (MMP)13* was considered a direct target of miRNA-125b
9	Olasz et al. [67]	upregulated	miRNA-135b	tissue	human	miRNA-135b can regulate cell migration and tumor invasiveness in early stages of SCC progression and can act as an oncogenic miRNA in human keratinocytes
9	Zhou M et al. [68]	upregulated	miRNA-365	tissue	human	downregulates *NFIB* (nuclear Factor I B) and inhibits the expression of cyclin-dependent kinase CDK6 and CDK4

**Table 3:** Modified miRNAs in MCC.

Nr	Ref	Expression	miRNA	Probe	Species	Role
1	Ning et al. [73]	upregulated	miRNA-9 miRNA-502-3p miRNA-7 miRNA-340 miRNA-182 miRNA-190b miRNA-873 miRNA-183	tissue	human	increasing tumor motility and colony formation. Potential diagnostic and therapeutic applications in cases of MCPyV-positive MCC
		downregulated	miRNA-3170 miRNA-125b miRNA-374c			not mentioned
2	Renwick et al. [74]	upregulated	miRNA-205 miRNA-375	tissue	human	microRNAs downregulate the expression of gene targets through interaction with their three prime untranslated region (3′ UTR). miRNA-375 targets *MTPN*(myotrophin) gene, which encodes the myotrophin protein, further regulating hormone release and exocytosis. Potential diagnostic role by discerning BCC from MCC
3	Veija et al. [76]	downregulated	miR-34a, miR-1539, miR-30a, miR-142-3p	tissue	human	may play a role in the oncogenesis of MCV-negative tumors.
		upregulated	miR-181d			

## Data Availability

Not applicable.
